# Application of a novel M-NED in ENBD patients: A case series report

**DOI:** 10.1097/MD.0000000000033215

**Published:** 2023-03-10

**Authors:** Zhaohui Liu, Runhua Lin, Ruinuan Wu, Jiwen Deng, Dayong Sun

**Affiliations:** a The Department of Gastroenterology, Shenzhen Second People’s Hospital, the First Affiliated Hospital of Shenzhen University Health Science Center, Shenzhen, China; b The Department of Gastroenterology, First Affiliated Hospital of Soochow University, Suzhou, China; c Department of Pathology, Shantou University Medical College, Shantou, China; d Guangdong Provincial Key Laboratory of Infectious Diseases and Molecular Immunopathology, Shantou, China; e The Department of Pathology, Shenzhen Second People’s Hospital, the First Affiliated Hospital of Shenzhen University Health Science Center, Shenzhen, China; f Shenzhen University School of Medicine, China.

**Keywords:** adverse events, ENBD, mouth-nose exchange, novel M-NED

## Abstract

The traditional methods for exchanging the endoscopic nasobiliary drainage (ENBD) tube from the mouth to the nose, such as the guidewire method, sponge holding forceps method, and finger method, have significant drawbacks including pharyngeal stimulation symptoms, high incidence of epistaxis, low success rate, and potential bite injuries to the operator.

**Patient concerns:**

A case series of 9 patients who underwent ENBD at Shenzhen Second People’s Hospital from January 2021 to December 2021 was collected.

**Diagnoses:**

The study included 9 patients diagnosed with choledocholithiasis, with 3 males and 6 females, with an average age of (55 ± 9.798) years (range 43–71).

**Interventions:**

The M-NED was used to exchange the ENBD tube, and the success rate, operation time, and complications were recorded.

**Outcomes:**

All patients successfully completed the operation in one go with an average mouth-nose exchange time of (44.67 ± 13.388) seconds (range 28–65). Two patients had mild adverse events, one of which was controllable bleeding caused by nasal mucosal injury with an estimated blood loss of 1 mL. The other patient had nausea during the operation, which improved after completion.

**Lessons:**

The novel M-NED is an effective and safe method for exchanging the ENBD tube from the mouth to the nose with a high success rate and low incidence of complications. It is a device with potential clinical application value.

## 1. Introduction

Endoscopic nasobiliary drainage (ENBD) is a commonly used procedure for draining bile from the bile duct under endoscopy.^[1]^ It is performed to relieve bile duct obstruction after endoscopic retrograde cholangiopancreatography (ERCP) and to prevent bile duct infections.^[2,3]^ However, the external end of the ENBD tube remains in the patient’s oral cavity, causing discomfort and affecting the patient’s bite.

To address these issues, different exchange methods have been used in clinical practice, including the guidewire method, sponge-holding forceps method, and finger method.^[4]^ However, these methods have been found to cause significant symptoms of pharyngeal stimulation, a high rate of bleeding in the nasal mucosa, and a low success rate. Additionally, there is a risk of operator fingers being bitten by the patients.

To overcome these problems, our research team has developed a new mouth-nose exchange device (M-NED). The M-NED is designed to be in line with the anatomy of the nasal cavity and to achieve the goal of folding back from the pharynx nasalis and then extending out of the mouth through the incisors. This paper reports on the clinical effects of using the M-NED for ENBD.

## 2. Materials and Methods

### 2.1. Study design and ethics

From January 2021 to December 2021, a retrospective analysis was conducted on all patients who underwent M-NED procedure after ENBD at our center. All patients provided informed consent for the surgery, and the study was approved by the Ethics Committee of the Second People’s Hospital of Shenzhen City.

### 2.2. Patients

All patients complained of abdominal pain. They were diagnosed with choledocholithiasis through magnetic resonance cholangiopancreatography. Informed consent was obtained from the patients for the publication of this case’s report details.

### 2.3. Instruments

The following instruments and equipment were used: image processing device (Olympus, Japan, CLV-290SL), electronic duodenoscope (Olympus, Japan, TJF 260V), high-frequency electrosurgical knife (ERBE, Germany, VIO300D), and disposable nasobiliary drainage catheter (Olympus, Japan, BD-24Z).

### 2.4. M-NED procedure

**Figure 1.**
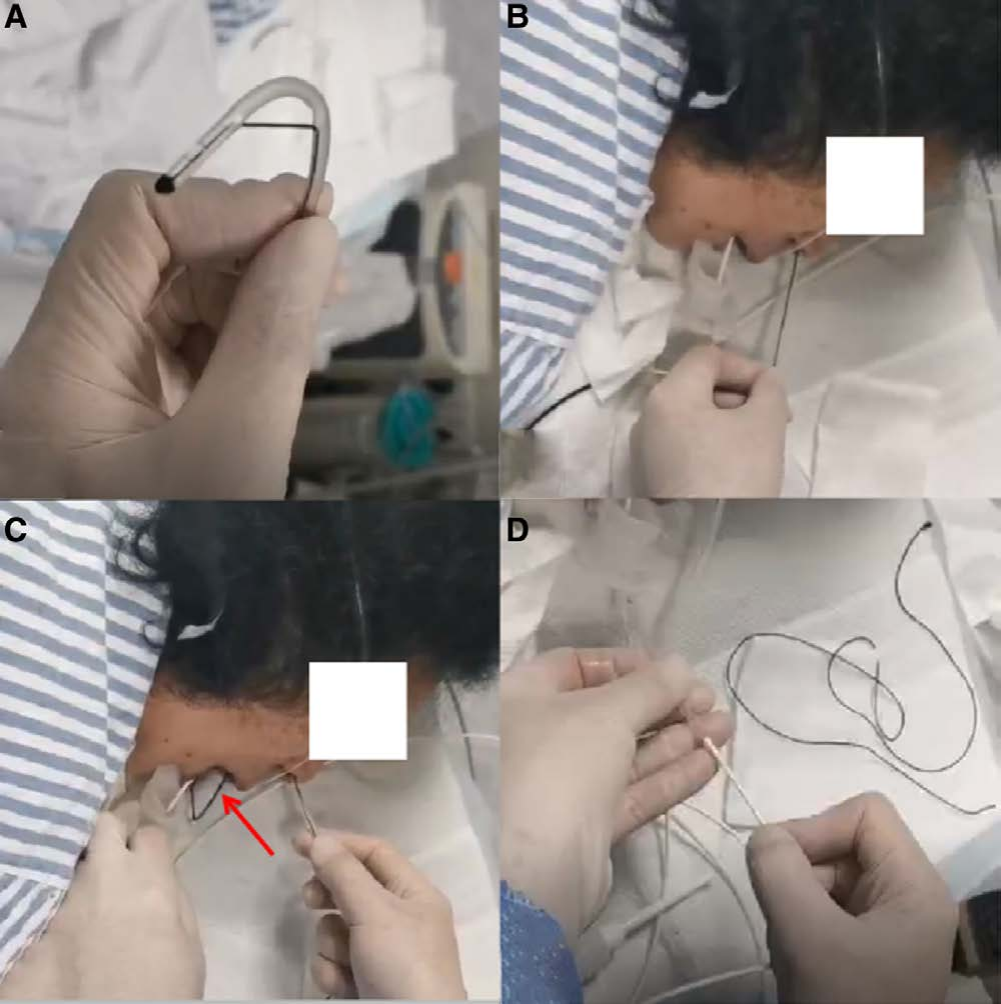
Procedure for using the M-NED on a patient. (A) The front end is adjustable and bendable. (B) The M-NED is inserted into one side of the nostril. (C) The front end of the M-NED exits through the mouth (indicated by the red arrow). (D) The external part of the ENBD tube is connected to the M-NED and pulled through the nasal cavity to complete the exchange. ENBD = endoscopic nasobiliary drainage, M-NED = mouth-nose exchange device.

**Figure 2.**
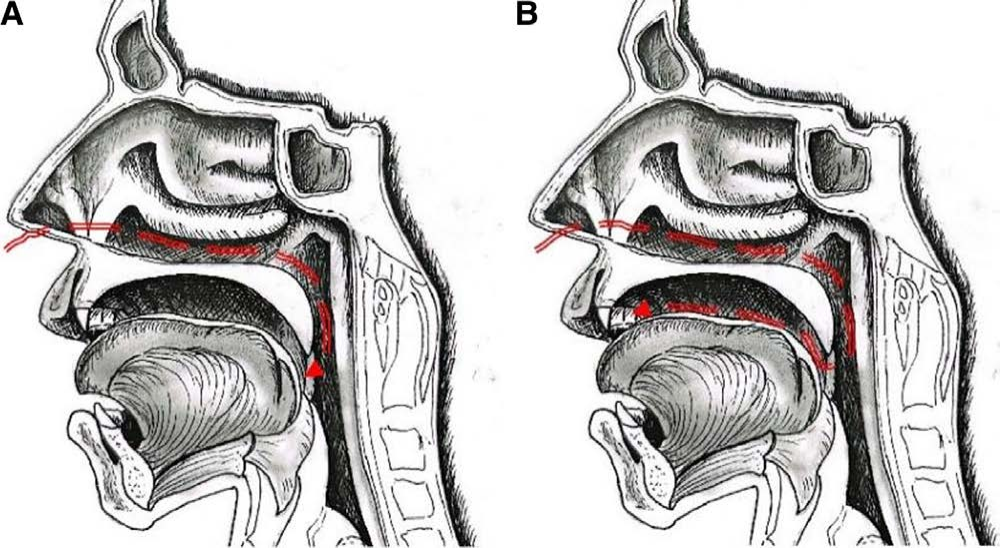
M-NED operation simulated diagram. (A) Insert the M-NED into the inner nostril and bend the front end in reverse. (B) Slowly insert the M-NED until the end of the tube is visible in the oral cavity. M-NED = mouth-nose exchange device.

The M-NED was lubricated before use. The front end of the M-NED was designed with a curved shape (Fig. 1A) to ensure it traveled in the direction of the lower nasal passage when inserted into the nasal cavity (Fig. 1B). As the M-NED was advanced to a depth of approximately 8 cm (Fig. 2A), it was gradually bent towards the oral cavity and then slowly straightened as it was inserted further until the end of the M-NED tube was visible in the oral cavity (Figs. 1C and 2B). The ENBD tube was then exchanged with the M-NED (Fig. 1D).

### 2.5. Observation indicators

The operation time was recorded as the time from the entry of the M-NED into the external naris to its exit from the incisor. Nasal bleeding was defined as any visible bleeding macroscopically.

### 2.6. Statistical analysis

Statistical analyses were performed using SPSS version 28.0 (IBM Corp, Armonk, NY). A single-sample *t* test was used to analyze the data for age (years) and operation time (seconds).

## 3. Results

Nine patients, including 3 males and 6 females, were included in the study with an average age of (55 ± 9.798) years (range 43–71). All patients underwent successful exchange of the ENBD tube from the oral cavity to the nasal cavity once, with an average exchange time of (44.67 ± 13.388) seconds (range 28–65). Two patients experienced complications during the exchange, with 1 minor bleeding due to nasal mucosal injury (estimated 1 mL bleeding) and the other with nausea which resolved after the exchange. No severe adverse events occured in any of the patients (Table 1).

**Table 1:** Population demographics and results.

Patient no.	Sex (M/F)	Age (yr)	Primary disease	Operation time (s)	Completion (yes/no)	Complications
1	F	43	Choledocholithiasis	53	Yes	No
2	F	47	Choledocholithiasis	47	Yes	No
3	F	51	Choledocholithiasis	65	Yes	No
4	M	68	Choledocholithiasis	47	Yes	Yes (bleeding)
5	F	46	Choledocholithiasis	30	Yes	No
6	F	71	Choledocholithiasis	28	Yes	No
7	M	54	Choledocholithiasis	61	Yes	No
8	M	54	Choledocholithiasis	38	Yes	Yes (nausea)
9	F	61	Choledocholithiasis	33	Yes	No
Mean		55		44.67		
SD (±)		9.798		13.388		
Maximal value		71		65		
Minimum value		43		28		

F = female, M = male, SD = standard deviation.

## 4. Discussion

ERCP has been used for nearly 70 years as a diagnostic and therapeutic tool in treating various biliary and pancreatic diseases, such as stones, tumors, and benign or malignant strictures.^[1,5,6]^ ENBD is a treatment technique developed from ERCP and has been shown to be effective in relieving biliary obstruction, controlling and preventing biliary infections, and decompressing bile drainage.

The advantages of ENBD include real-time monitoring of the drainage volume and color, which helps evaluate the recovery of the bile duct and early detection of complications.^[7]^ Currently, the focus of clinical attention is on fixing the anti-slip ENBD tube,^[8]^ and there is limited research on the mouth-nose exchange of the ENBD tube. The commonly used methods are the finger method, sponge-holding forceps method, and guidewire method.^[4]^ However, in a significant number of clinical practices, we have found that many problems often occur during the exchange of the ENBD tube from the oral cavity to the nasal cavity, such as nasal bleeding, failure to withdraw the nasal catheter from the oral cavity, discomfort, and sometimes biting the operator’s fingers. Therefore, it is critical to find a safe and effective method to solve these problems. Currently, there are no related research reports to address these adverse events.

Our team has designed a new M-NED (Chinese Patent, Patent No. ZL20212344880.6), which has a folding function at the front end and is anatomically structured during the operation, allowing the front end of the M-NED to be delivered outside the oral cavity without any other assistance methods. Theoretically, this is a more effective way. In this study, by reviewing the clinical data of 9 patients, the results showed that 9 cases (100%) were successfully completed with mouth-nose exchange.

The common adverse events associated with the ENBD mouth-nose exchange procedure include nasal bleeding, strong discomfort, and sometimes biting of the operator’s fingers by patients. In this study, 1 patient had a minor episode of nasal bleeding with an estimated volume of 1 mL, which resolved spontaneously without any special intervention. Another patient experienced nausea during the procedure, which was believed to be caused by the stimulation of the pharyngeal posterior wall by the M-NED. However, nausea disappeared after the procedure was completed. No severe adverse events were reported in any of the patients.

There is no data available on the average operation time for the currently used ENBD exchange methods. However, based on clinical experience, longer operation times increase the risk of adverse events, such as discomfort in the patients, including nausea, vomiting, pharyngeal pain, etc. In addition, repeated operations also increase the risk of nasal mucosal damage. The results of this study showed that the average time to complete the ENBD exchange was (44.67 ± 13.388) seconds, with a maximum operation time of 65 seconds and a minimum operation time of 28 seconds, which indicates a relatively short overall operation time.

According to the present study, the operation of M-NED has a high success rate with minimal adverse events and a short duration, which may be attributed to its unique design. The adjustable bend structure at the front end is able to effectively reduce the likelihood of nasal bleeding by adapting to the contours of the nasal canal during insertion. The reverse fold design at the inner nose hole guides the M-NED towards the incisor, increasing the success rate of the procedure. Additionally, the absence of guide wires and fingers reduces the strong stimulation to the pharynx, leading to a noticeable reduction in patient discomfort.

The primary drawback of the present study is its limited design as a retrospective, single-center observational study with a small sample size. However, this does not hinder the ability to draw firm conclusions about the effectiveness and safety of M-NED.

In conclusion, M-NED is a novel type of ENBD M-NED that has demonstrated both safety and efficacy in this study.

## Acknowledgment

The authors would like to acknowledge Mrs Xia Lei for her contribution to creating Figure 2 for this publication.

## Author contributions

**Data curation:** Jiwen Deng.

Formal analysis: Runhua Lin.

Funding acquisition: Zhaohui Liu.

Methodology: Zhaohui Liu.

Project administration: Dayong Sun.

Supervision: Ruinuan Wu.

Writing – original draft: Zhaohui Liu.
